# Draft Genome Sequence of Sweet Pepper Fruit Epiphyte-Associated Bacillus cereus HRT7.7

**DOI:** 10.1128/mra.01125-21

**Published:** 2022-02-10

**Authors:** Tshifhiwa P. Mamphogoro, Casper N. Kamutando, Martin M. Maboko, Olayinka A. Aiyegoro, Olubukola O. Babalola

**Affiliations:** a Gastro-Intestinal Microbiology and Biotechnology Unit, Agricultural Research Council-Animal Production, Irene, Pretoria, South Africa; b Department of Plant Production Sciences and Technologies, University of Zimbabwe, Harare, Zimbabwe; c Department of Crop Sciences, Tshwane University of Technology, Pretoria, South Africa; d Research Unit for Environmental Sciences and Management, North-West University, Potchefstroom, South Africa; e Food Security and Safety Niche Area, Faculty of Natural and Agricultural Sciences, North-West University, Mmabatho, South Africa; Montana State University

## Abstract

This study reports the whole-genome sequence of Bacillus cereus HRT7.7, an epiphyte isolated from red sweet pepper fruits that is capable of stimulating plant growth and development. The genome assembly is 5,109,010 bp in length, with a G+C content of 35.2%.

## ANNOUNCEMENT

Epiphytes are microbes that colonize and multiply nonparasitically on the surfaces of plant organs (1, 2). They frequently display symbiotic relationships with plants. Plants provide epiphytes with a medium for growth and survival, and in return some epiphytes stimulate plant growth and development through functional roles such as phytohormone production, nitrogen fixation, and solubilization of phosphorus and iron. In addition, some epiphytes antagonize the activity and proliferation of pathogenic microbial strains (3–6).

Bacillus cereus HRT7.7 was isolated from the surface of sweet pepper fruit samples. The samples were sourced from the Agricultural Research Council-Vegetable and Ornamental Plants (ARC-VOP), Roodeplaat, Pretoria, South Africa (25°59′S, 28°35′E). Fresh, matured red sweet pepper samples (treated with fungicides) grown in a hydroponic cropping system were aseptically collected in sterile zipper-lock bags, transported to the laboratory, and stored at 4°C (7). Bacterial biofilms on the surfaces of the pepper fruits were recovered using sterile swabs soaked in a solution containing 0.15 M NaCl and 0.1% (vol/vol) Tween 20 (8). The swabs were then vortex-mixed in sterile Eppendorf tubes containing physiological saline solution, the supernatant was serially diluted up to 10^−2^, and 100-μL aliquots were cultured on Trypticase soy agar (TSA) plates. The plates were incubated for 48 h at 30°C under aerobic conditions. A pure culture was obtained by repeated streaking on sterile TSA, incubated at 30°C for 48 h, and stored in 50% (vol/vol) glycerol at −80°C for further use.

Purified B. cereus HRT7.7 in 50% glycerol at −80°C was subcultured on TSA and then used for DNA extraction. Genomic DNA was extracted from an overnight culture by employing a fungal/bacterial miniprep kit (Zymo Research, Irvine, CA, USA), following the manufacturer's instructions. The concentration of the extracted DNA was measured using a NanoDrop spectrophotometer (Thermo Fisher Scientific, Carlsbad, CA, USA), while the DNA quality was evaluated on a 2% agarose gel. The strain was taxonomically identified by analyzing the 16S rRNA sequence using BLASTn v2.12.0 (9, 10). DNA libraries were generated using a NEBNext Ultra II FS DNA library preparation kit (New England Biolabs, MA, USA) and sequenced with a paired-end sequencing strategy (2 × 150 bp) using the Illumina NextSeq 550 platform, at a commercial service provider (Inqaba Biotechnical Industries [Pty] Ltd., Pretoria, South Africa). The sequences were analyzed on the KBase platform (11). The quality of the reads was evaluated using FastQC v0.11.5 (12), while removal of sequence adaptors and low-quality reads (quality scores of <15) was performed with Trimmomatic v0.36 (parameters, ILLUMINACLIP:TruSeq3-PE.fa:2:30:10 SLIDINGWINDOW:4:15 MINLEN:75) (13). Reads were assembled with SPAdes v3.13.0 in the –careful mode (14), and the procedure yielded a total of 219 contigs, with an average of 280-fold coverage.

HRT7.7 yielded a genome of 5,109,010 bp, with a G+C content of 35.2%. The contigs showed *N*_50_ and *L*_50_ values of 302,853 bp and 6, respectively. Gene annotation was performed using the publicly available NCBI Prokaryotic Genome Annotation Pipeline (PGAP) (15) and predicted a total of 5,368 genes, including 5,167 protein-coding genes, 84 RNA genes, 12 rRNA genes, 67 tRNA genes, 117 pseudogenes, and 5 noncoding RNA genes. All analyses were performed using default parameters unless otherwise stated. Secondary metabolites were determined by antiSMASH v6.0.0 (16), and this strategy identified genes responsible for plant hormone production, transcriptional regulators, transport proteins, and nitrogen fixation, all of which play a significant role in symbiosis with the plant and promote plant growth and development (17, 18).

### Data availability.

This whole-genome shotgun project and associated data have been deposited in DDBJ/ENA/GenBank under the accession number JAJFEX000000000, BioProject accession number PRJNA771517, BioSample accession number SAMN22314523, and SRA accession number SRX12629285. The version described in this paper is JAJFEX000000000.1.
